# Quadruple Valve Replacement in Carcinoid Heart Disease: A Case Report

**DOI:** 10.7759/cureus.45547

**Published:** 2023-09-19

**Authors:** Maria Beyer, Rishabh Kasarla, Seth Shoap, Erik Beyer

**Affiliations:** 1 Department of Cardiothoracic Surgery, Nova Southeastern University Dr. Kiran C. Patel College of Allopathic Medicine, Fort Lauderdale, USA; 2 Department of Cardiothoracic Surgery, Palmetto General Hospital, Hialeah, USA

**Keywords:** carcinoid-triggered fibrosis, valvular prosthesis, valvular fibrosis, quadruple valve replacement, carcinoid heart disease

## Abstract

This report details a rare case of left-sided carcinoid heart disease (CHD). In CHD, vasoactive substances released from carcinoid tumors cause fibrous tissue formation on the right side of the heart. These substances are usually inactivated by monoamine oxidase A in the lungs, safeguarding the left side of the heart. Exceptions include the presence of a patent foramen ovale (PFO), pulmonary metastasis, or elevated serotonin levels. Intriguingly, our patient exhibited significant left-sided involvement without these factors, ultimately requiring a quadruple valve replacement surgery. After eight months post-operation, the patient is stable with no cardiovascular complications. This rare case of CHD, along with its outcome, hints at potential unidentified etiologies for left-sided CHD and underscores valve replacement as a viable treatment.

## Introduction

Carcinoid tumors, a rare subset of neuroendocrine tumors, can cause carcinoid heart disease (CHD) through the release of vasoactive substances such as serotonin, histamine, bradykinins, prostaglandins, and tachykinins [1,2]. These vasoactive substances are also responsible for the characteristic symptoms seen in carcinoid syndrome, including skin flushing, bronchospasm, and gastrointestinal hypermotility. In CHD, these substances induce fibrous tissue formation on heart valves, typically on the right side, causing heart failure [3]. Involvement of the left side is rare, as vasoactive substances secreted by the neuroendocrine tumor are inactivated by monoamine oxidase A in the lungs [3]. However, left-sided CHD can occur, usually with a patent foramen ovale (PFO), pulmonary metastasis, or high serotonin levels. It is through these means that serotonin released by the tumor can divert inactivation in the lungs and access the left heart. Previous cases vary in presentation and treatment but generally necessitate surgical intervention for valve repair or replacement. The case we discuss is novel as it required quadruple valve replacement, with no identified PFO or pulmonary metastasis, emphasizing the significance of left-sided CHD and valve replacement.

## Case presentation

**Figure 1:**
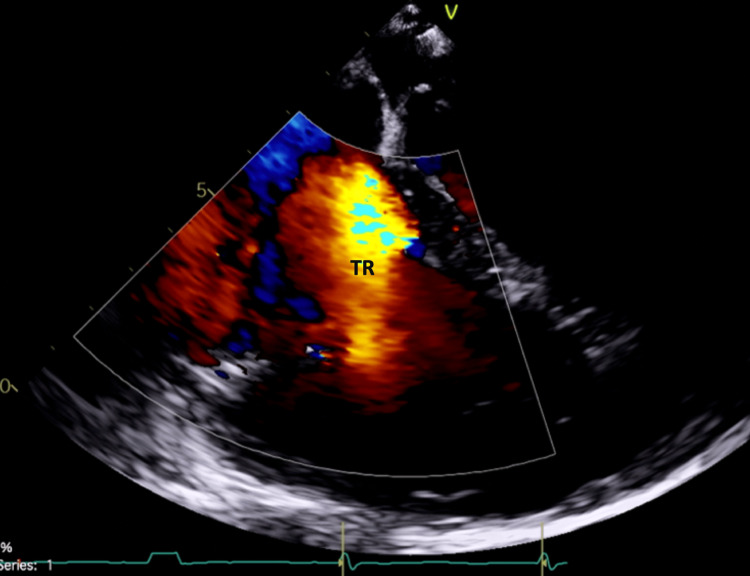
Echocardiogram indicating severe TR prior to valve replacement TR: tricuspid regurgitation

**Figure 2:**
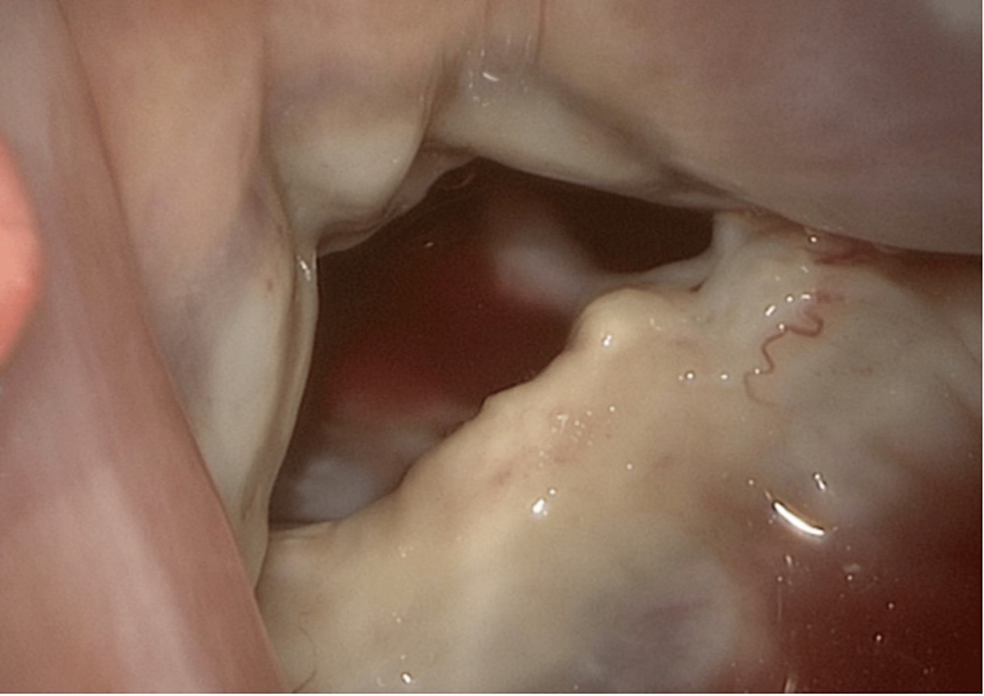
Tricuspid valve prior to valve replacement showing retracted and fibrotic leaflets

**Figure 3:**
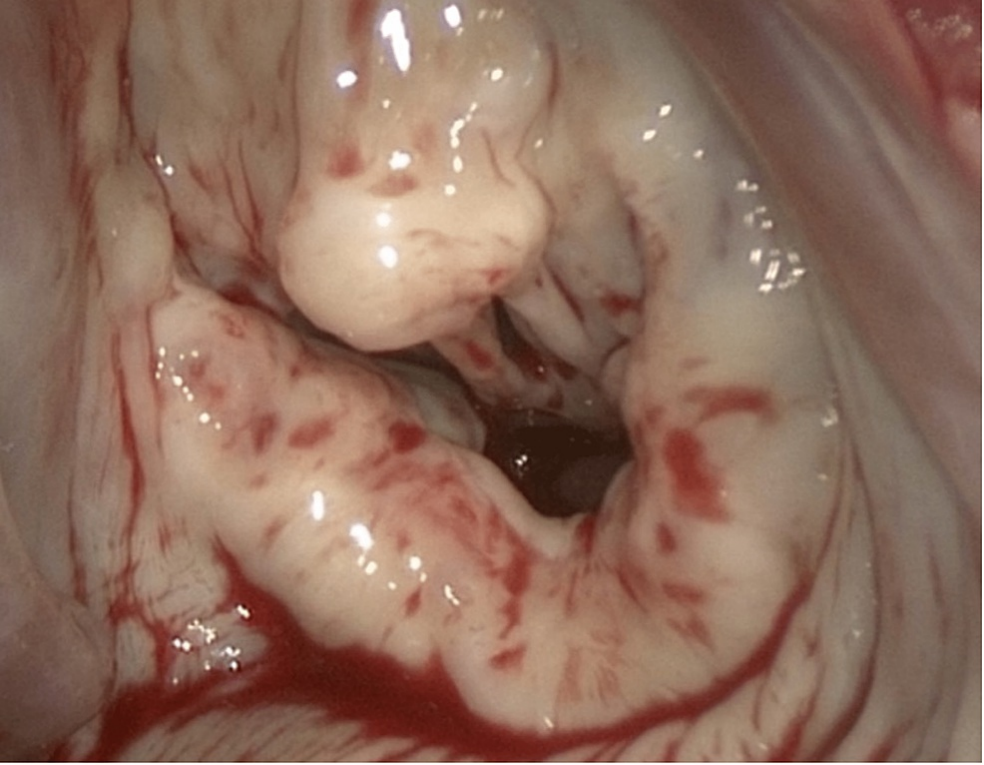
Carcinoid-triggered fibrosis led to significantly damaged valves The mitral valve is shown.

A 64-year-old female with a cecal carcinoid tumor history, hemicolectomy, and severe tricuspid regurgitation came in with chest pain and dizziness. Clinical findings included a heart rate of 70 bpm, respiratory rate of 18 breaths per minute, and blood pressure of 104/71 mmHg. Laboratory results suggested prerenal azotemia, likely from decreased cardiac output. Chest computed tomography (CT) and transesophageal echocardiogram (TEE) unveiled a severely dilated right ventricle, 4+ tricuspid (Figure 1), pulmonic regurgitation due to right-sided leaflet restriction and retraction (Figure 2), and significant left-sided leaflet thickening (Figure 3) with 3+ mitral regurgitation and aortic insufficiency. Given the severity of heart involvement, surgery was the chosen treatment.

The surgical procedure involved sequential replacement of all four valves under bypass (Video 1). The mitral valve was replaced with a porcine bioprosthesis through a left atrial transseptal incision. A right atrial incision was made to replace the tricuspid valve with a pericardial tissue valve. The pulmonic valve underwent a similar replacement procedure, followed by right ventricular outflow tract (RVOT) reconstruction. After an aortotomy, the aortic valve was replaced with a pericardial tissue valve. Each step required precise incisions, valve visualization, replacement, and closure. Postoperative care was centered on monitoring vitals, pain management, and vigilance for complications.

**Video 1:** Quadruple valve replacement in carcinoid heart disease The surgical procedure involved sequential replacement of all four valves under bypass. Each step required precise incisions, valve visualization, replacement, and closure.

On postoperative day 6, the patient was discharged. Laboratory results showed normalized blood urea nitrogen (BUN) and creatinine, and increased red blood cell (RBC), hemoglobin (Hgb), and hematocrit (Hct) levels compared to admission. At her one-month follow-up, she exhibited excellent functional status, significant heart failure symptom improvement, and absence of ascites. Presently, eight months post-surgery, she maintains a stable condition with no cardiovascular complications.

## Discussion

Our approach to this case was characterized by a comprehensive consideration of the patient's symptoms and medical history. Dizziness, constant chest pain, and notable cardiac abnormalities on clinical examination, coupled with a known history of cecal carcinoid tumor, steered us toward suspecting a recurrence of carcinoid syndrome manifesting as CHD [2]. Additionally, the collaborative effort of our interdisciplinary team of specialists including cardiologists, cardiothoracic surgeons, and oncologists allowed for effective management of this complex case. However, our approach was limited by the lack of established guidelines or protocols for managing such a rare presentation of CHD affecting all four heart valves.

While most existing literature emphasizes right-sided valve involvement in CHD, due to the typical metastasis route in carcinoid syndrome, cases presenting left-sided CHD are less frequent. Additionally, a case involving all four valves, without a patent foramen ovale or pulmonary metastasis, as observed in our patient, is exceptionally rare. The patient's case suggests the possibility of undiscovered etiologies of left-sided CHD, inviting further investigation. Previously reported cases of left-sided carcinoid syndrome have shown variability in both the extent of the disease and the treatments used. For instance, Lichtenauer et al. documented a case with minor mitral valve sclerosis, which was managed by surgical reconstruction of the valve [4]. On the other hand, the research by Connolly et al. indicated that about half of their patient pool required replacement or repair of mitral and aortic valves due to left-sided CHD, despite variations in disease extent and etiology [1]. Our case report highlights an unconventional scenario of quadruple valve replacement.

## Conclusions

The patient's stable condition and lack of cardiovascular complications eight months post-operation indicate a successful outcome. This unusual case of left-sided CHD with successful quadruple valve replacement offers valuable insights into potential unknown etiologies and reinforces valve replacement as an effective treatment strategy. Further research into this area could prove instrumental in improving the understanding and management of CHD. This case illustrates that quadruple valve replacement can be successful in managing CHD affecting all four valves. This finding prompts the medical community to consider similar interventions for future cases.
